# Accurate THz ellipsometry using calibration in time domain

**DOI:** 10.1038/s41598-022-10804-w

**Published:** 2022-05-05

**Authors:** Zahra Mazaheri, Can Koral, Antonello Andreone

**Affiliations:** 1 Department of Physics “E. Pancini”, University of Naples “Federico II”, 80126 Naples, Italy; 2 INFN, Naples Unit, 80126 Naples, Italy

**Keywords:** Optics and photonics, Physics

## Abstract

We report on the realisation of a customized THz time domain spectroscopic ellipsometer (THz-TDSE) based on fiber-coupled photoconductive antennas, operating in a wide range of incident angles and allowing also standard transmission spectroscopy without any optical realignment. To ensure accurate parameter extraction for a broad range of materials, we developed a fast and effective algorithm-assisted method to calibrate the setup and compensate for the nonideality in the response of the THz system. The procedure allows to minimise errors induced by imperfect response of the antennas and polarizers, imprecise setting of the impinging and receiving angles in the goniometric mechanical arms, and unavoidable mismatches in the THz beam optics. Differently from other calibration methods applied in the literature, our approach compares in time domain the ellipsometric derived electric field s- and p-polarised components at a given angle of incidence with the reconstructed ones, attained by using the complex dielectric function of a known sample. The calibrated response is determined with high precision by setting the system in transmission mode. In order to validate the technique, ellipsometric measurements have been carried out at various angle of incidences on a number of materials both in solid and liquid form, and their data compared with what obtained by conventional THz spectroscopy. Results show that THz-TDSE accompanied with an accurate calibration procedure is an effective technique for material characterization, especially in case of samples with a high absorption rate that are not easily investigated through transmission measurements.

## Introduction

The terahertz (THz) frequency window lies in between the microwave and infrared bands^1^, with a corresponding energy in the range between $10^{-22}$ and $10^{-20}$ J, which is responsible for the onset of many important chemical and physical processes associated with vibrational and rotational motions at the molecular scale^2^. For this reason, any probing tool investigating this part of the electromagnetic spectrum provides information on specific signatures for many compounds^3^. Moreover, the harmless nature and the relatively large penetration depth of the THz radiation in different materials makes it suitable for non destructive testing and process control^4,5^.

Recently, spurred by the enormous improvement in the development of opto-electronic sources and detectors, spectroscopic characterization techniques started to thrive in this frequency range. The increasing interest in THz spectroscopy lies in its potential for providing accurate and precise deduction of the electro-optical properties for a wide range of materials with diverse properties^6^, which in turn leads to a better understanding of the operating principles of complex THz systems and devices^7^. Any significant progress in THz technologies for use in novel sensing and communication systems cannot leave out a concurrent advancement in the characterization techniques to determine how different materials, components, and systems respond to the impinging radiation^8^.

A key feature of the THz spectroscopy is that in this frequency band waves are able to penetrate a large number of organic or inorganic materials such as polymers, dielectrics, and semiconductors, which are opaque in visible or IR ranges^9^. Conversely, THz radiation is strongly absorbed by polar liquids.

In standard THz time domain spectroscopy (THz-TDS) the transient electric field in transmission or reflection mode is detected, and from here the complex refractive index of the sample can be directly determined. Material parameter extraction is a straightforward method in transmission geometry, since it involves the simple comparison of the time-dependent electric field passing through the sample with respect to a reference signal (transmitted in free space or across a substrate). However, in highly doped semiconducting and/or conductive materials the method is limited with the skin depth of the material, therefore it cannot be applied in case of bulk and/or optically thick samples. Similarly, it is not possible to use this technique for highly absorptive media such as biological systems or advanced multilayered structures composed of optically thin films grown on thick substrates^10^.

In principle for such non trivial systems THz reflection techniques can be applied, in practice however they highly suffer from technical limitations and uncertainties in sample and reference positioning and lead to a high uncertainty in the material parameter determination^11^. THZ-TDSE is a potentially strong candidate to overcome these obstacles.

Ellipsometry is a widely used and well established characterization technique in the optical regime. The change in the polarization states of light upon reflection from the sample under investigation is recorded by utilizing a continuously rotating analyzer. Given an incident signal reaching the sample surface at a predetermined angle $\theta$, how the electric field polarization states are modified after reflection directly correlates with the intrinsic dielectric properties of the material. The change in polarization states is expressed through the ellipsometric complex parameter $\rho$^12^:

1 $$\begin{aligned} \rho =\tan {\Psi } exp(i\Delta ) = \frac{r_{p}}{r_{s}} \end{aligned}$$

where $r_{p}$ ($r_{s}$) is the complex reflection coefficient for the p-polarised (s-polarised) component. Afterwards, the ellipsometric angles ($\Psi$, $\Delta$) are calculated by using Jones matrix analysis^13^ to extract the complex dielectric function of the material^14^. For a well calibrated system, further information on thickness and surface roughness can be estimated by means of suitable models^15^.

Due to its self-reference nature, ellipsometry is less prone to errors stemming from reference and sample relative misplacement. Because of the different physical processes involved in the THz region, expanding the ellipsometry operational frequency window to smaller frequencies allows to retrieve a wider range of information on complex phenomena like spin oscillations^16^, anti-ferromagnetic resonances^17^, transport in ferroelectrics^18^, free charge carrier oscillations^19^, and to fully characterize active and passive polarization sensitive devices such as metasurfaces^20^. Additionally, THz ellipsometry might successfully operate as a characterization technique for the study of challenging materials like polar fluids or highly conducting thin films and multilayers grown on a dielectric substrate.

In time domain THz spectroscopic systems the reflected signal is *coherently* detected, that is information on both intensity and phase of the reflected signals is retrieved, thereby allowing for the direct measurements of the ellipsometric parameters. Basically, an electric field pulse with approximately ps duration and known polarization is emitted and used to illuminate the sample. Then, the temporal profile of the (orthogonal) p- and s-polarised beam components reflected from the surface are separately detected. By transforming the time domain data related to each polarization state into the frequency domain through Fast Fourier Transform (FFT), and from the knowledge of the amplitude $r_{p}$ ($r_{s}$) and phase $\phi _{p}$ ($\phi _{s}$) of the p-polarised (s-polarised) component in the incident and reflected beam, one can extract in a straightforward manner the ellipsometric angles: $\tan {\Psi }=\frac{|r_{p}|}{|r_{s}|}$; $\Delta =\phi _{p}-\phi _{s}$.

These two parameters can be measured over a frequency range and then converted into material properties, like the refractive index *n* and extinction coefficient *k*. Equations (2) and (3) show the relation between $\Psi$ and $\Delta$ and the complex index of refraction $n + ik$ for the specific case of a homogeneous and optically thick sample^21^:

2 $$\begin{aligned} n^2-k^2&= \sin ^2{ \theta }\left[ 1+\frac{ \tan ^2{\theta }(\cos ^2 {2\Phi }-\sin ^2 {2\Phi } \sin ^2{\Delta })}{(1+\sin {2\Phi } \cos {\Delta })^2}\right] \end{aligned}$$

3 $$\begin{aligned} 2nk&= \sin ^2{\theta } \frac{\tan ^2{\theta } \sin {4\Phi } \sin {\Delta }}{(1+\sin {2\Phi } \cos {\Delta })^2}, \end{aligned}$$

where $$tan\Phi =\frac{1}{tan\Psi }$$.

A major drawback of a THz ellipsometer is that the focusing and matching conditions among different electro-optical components (antennas, lenses, polarizers) are more sensitive than the corresponding system in the optical regime. This renders the instrumentation setup and the calibration procedure very challenging. Hofmann et al. first^22^ and Kühne et al.^23^ subsequently proposed wavelength tunable ellipsometers operating in the frequency domain 0.1–1.5 THz and having as radiation source a backward wave oscillator. The THz beam was detected by using bolometer detectors or a Golay cell. In their case therefore detection was non coherent indicating the need for a rotating element. The configuration used was a rotating analyzer scheme (source-polarizer-sample-analyzer-detector). A study from Neshat and Armitage^24^ reported a variable angle ($15^{\circ }<\theta <90^{\circ }$) setup having the emitter arm fixed and the detector arm free to rotate. The symmetry between the incident and reflected angles was provided by a rotating stage where the sample was placed. To take into account uncertainties in the polarization response and errors in the angular settings, they used a conventional regression calibration first proposed by B. Johs for rotating element optical ellipsometer^25^. Lately, Chen et al.^26^ proposed a robust ellipsometer operating in the range 0.1–1.6 THz and fiber-coupled in both arms, providing multi-angle measurements in the range of $45^{\circ }<\theta <90^{\circ }$. A complicated calibration algorithm based on interference theory was used to take into account pulse shift errors and limited extinction ratio of the polarizers. Very recently, Baez-Chorro et al.^27^ proposed a THz ellipsometric setup where, by using a birefringent medium, information on the two orthogonal polarizations was encoded in a single trace. Introducing this method, they eliminate the need of the analyzer overall simplifying measurements. The method however suffers from the fact that the time domain signal for each electric field component must be “windowed”, which limits the accuracy in the frequency response.

In the following, we report on the setup of a customised time domain ellipsometric system, operating in the range 0.1–0.6 THz and capable of accurate alignment in a wide range of incident angles ($20^{\circ }<\theta <90^{\circ }$). We show that, using an additional polarizer in the detector arm of the ellipsometer set-up and changing its orientation at each incident angle through an in-house developed algorithm, it is possible to compensate all the errors induced by optical mismatch or imperfections in polarisers and antennas response. The instrumentation allows also with ease standard transmission spectroscopic measurements without any setup realignment.

The fast and effective algorithm assisted compensation technique for the accurate calibration of the THz ellipsometer is described in detail. In order to validate the method, ellipsometric measurements at various angles of incidence are reported on different solid and liquid samples, having a dielectric permittivity ranging from relatively lower to much larger than the reference material (high resistivity Silicon) adopted for the calibration procedure. The retrieved complex dielectric properties of the samples under test are then compared with the same parameters obtained using the standard THz spectroscopy analysis.

## Methods

The custom designed ellipsometric system is based on a time domain spectrometer driven by an EKSPLA laser with pulse duration less than 150 fs and operating in free space at 1045 nm with 30 MHz pulse repetition rate. Fiber-coupled photoconductive antennas (PCA) are used for both THz emission and detection. A fast delay line is placed at the detection arm for rapid data acquisition and time domain signal averaging. A beam having Gaussian profile and waist less than 3 mm is driven to the PCAs through free space-to-fiber couplers (efficiency approximately $50\%$ for both emitter and detector arms) and hollow core photonic crystal fibers (HC-1060 from Thorlabs) with near zero dispersion at the laser wavelength. However the free space-fiber coupling unavoidably causes losses and dispersion in the laser beam, presently reducing the frequency range of the TDSE system below 1 THz. A standard optical setup with two different pairs of polymethypenetene (TPX) lenses is used to collimate and focus the THz signal emitted by the first PCA, impinging onto the sample plane and then received by the second PCA. To avoid angular divergence of the radiation, it is important to use the lens pair with larger *f*-number to concentrate on and to collect the THz signal from the sample. We use lenses with *f*-number 1.31 and 2.62 to collimate and focus the THz beam respectively. For the control and detection of the polarization states we employ three substrate free Wire Grid Polarizers (WGP) from PureWavePolarizers Ltd, with broadband transmission and high extinction ratio ranging between 20 and 50 dB. Each polarizer has wire diameter $10~\upmu \hbox {m}$, wire spacing $25~\upmu \hbox {m}$, an aperture of 15 mm, and is placed on a high precision computer controlled rotational stage. The first WGP (P) is placed in front of the emitter PCA guaranteeing that a perfectly linear polarized signal is reaching the sample surface. Along the detector arm, a second WGP (A) is placed to work as analyzer, to select the p or s polarization component of the beam upon reflection. Right after that, there is a third WGP (C) that ensures that detection has equal sensitivity to the p- and s-polarized signals and eventually compensate possible errors in setting their ratios.

**Figure 1:**
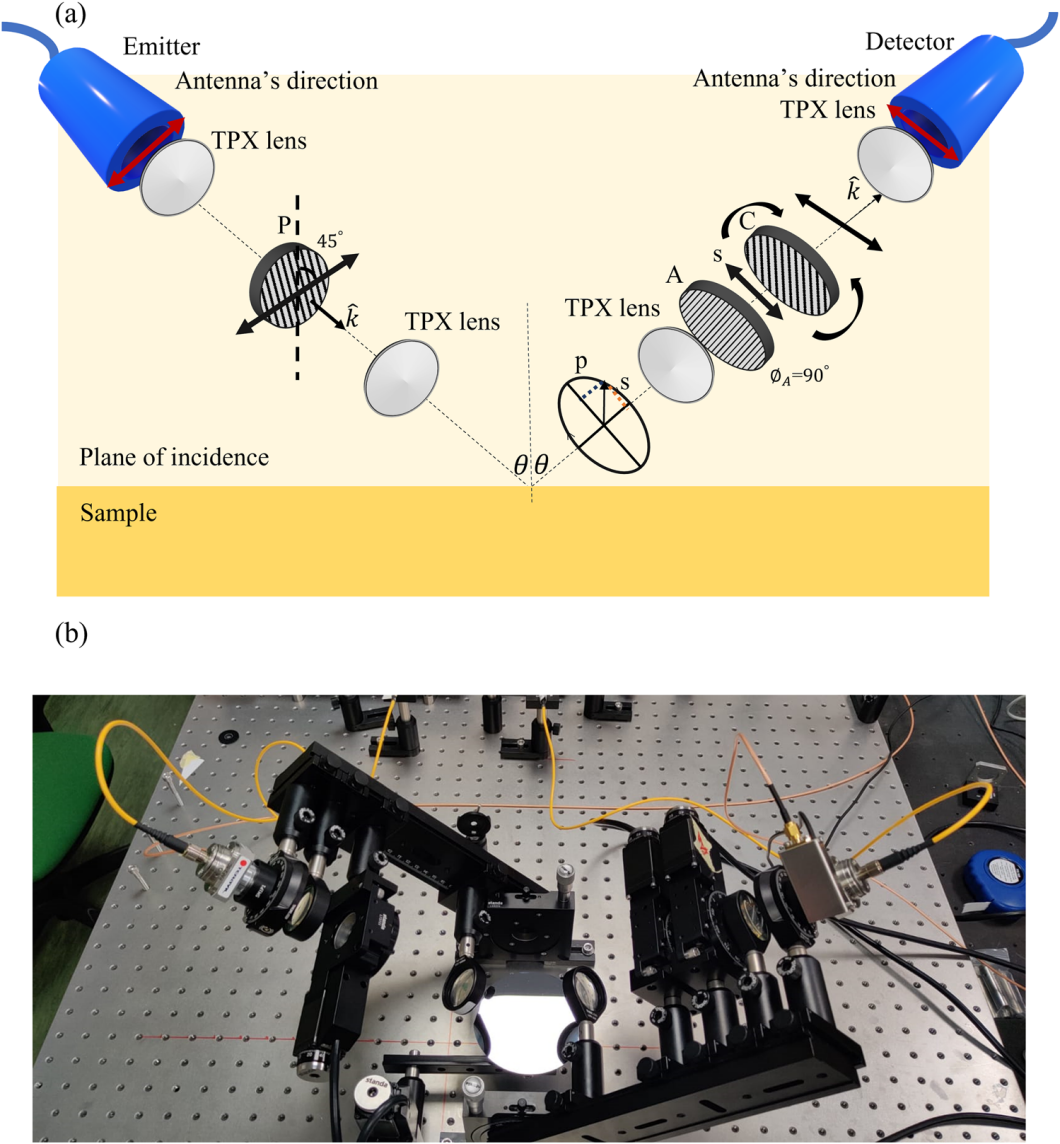
(**a**) Opto-mechanical setup sketch and (**b**) picture of the realised fiber-coupled THz-TDSE. In (**a**) *P*, *A*, *C* stand for the role played by each wire grid polarizer, and the scheme for the detection of the s-polarised component ($\phi _{A}=90^{\circ }$) is shown.

The opto-mechanical design combined with the fiber-coupled antennas makes the system flexible and capable to adjust the angle of incidence and reflection without any need for further alignment. It also allows to perform THz transmission measurement by only rotating the sample orientation at $90^{\circ }$. Figure 1 displays (a) the schematic representation along with (b) a picture of our customized THz ellipsometer. Following the definition, the electric field component parallel to the plane of incidence is p-polarized, the one perpendicular is s-polarized. Along the emitter arm, the photoconductive antenna that provides the linearly polarized THz radiation is rotated by $45^{\circ }$ in the azimuth plane. In this configuration, the eigenstates (p- and s-polarized) of the signal coming from the emitter are distinguishable. All azimuth angles are defined by the orientation with respect to the plane of incidence, clockwise being the positive rotation looking from the source to the detector. To remove any cross-polarization, polarizer P is therefore aligned along the electric field direction, with an azimuth angle $\phi _{P}$ set at $45^{\circ }$. Polarizer A is set at $\phi _{A}=0^{\circ }$ or $\phi _{A}=90^{\circ }$ depending on which reflected beam component, p- or s-polarized, must be selected. As mentioned earlier, the role of the polariser C is to modify in time domain the ratio of p- and s-polarized electric field component in order to compensate possible error produced by imperfect dipolar response of the photoconductive antennas, imprecise orientation of polarizers P and A, or incorrect settings of the incident and reflecting angle. Under ideal experimental conditions, the compensator azimuth angle should be kept at $\phi _{C}=-45^{\circ }$. In reality, this angle might change—depending also on the angle of incidence—in a small range centered around or close-by $-45^{\circ }$, and its value should be accurately determined.

The THz beam waist at the focus point is measured to be approximately 5 mm. The sample is placed on a kinematic platform coupled with two-axis motorised linear stages to achieve a sensitive control of the focusing conditions and surface parallelism with respect to the beam wavefront. Although ellipsometry is less sensitive to an imprecise sample positioning, its accurate placement at the focal length enhances the detection of the signal reflected from the surface^23^.

Data acquisition is realized by means of a lock-in amplifier coupled with electronics and computer software. Time domain signals, averaged over 500 waveforms, are recorded in a temporal interval of 120 ps and then converted into the frequency domain by FFT. The configuration of this ellipsometer allows also to measure biological and liquid samples with ease and in open sample holders since the arms are designed to move in a plane perpendicular to the optical table (see Fig. 1b).

In ellipsometric measurements, calibration is an essential albeit time consuming step to enhance material characterization accuracy. To achieve an effective, fast and precise calibration, we pursue some strategies that are explained in details in the following.

**Figure 2:**
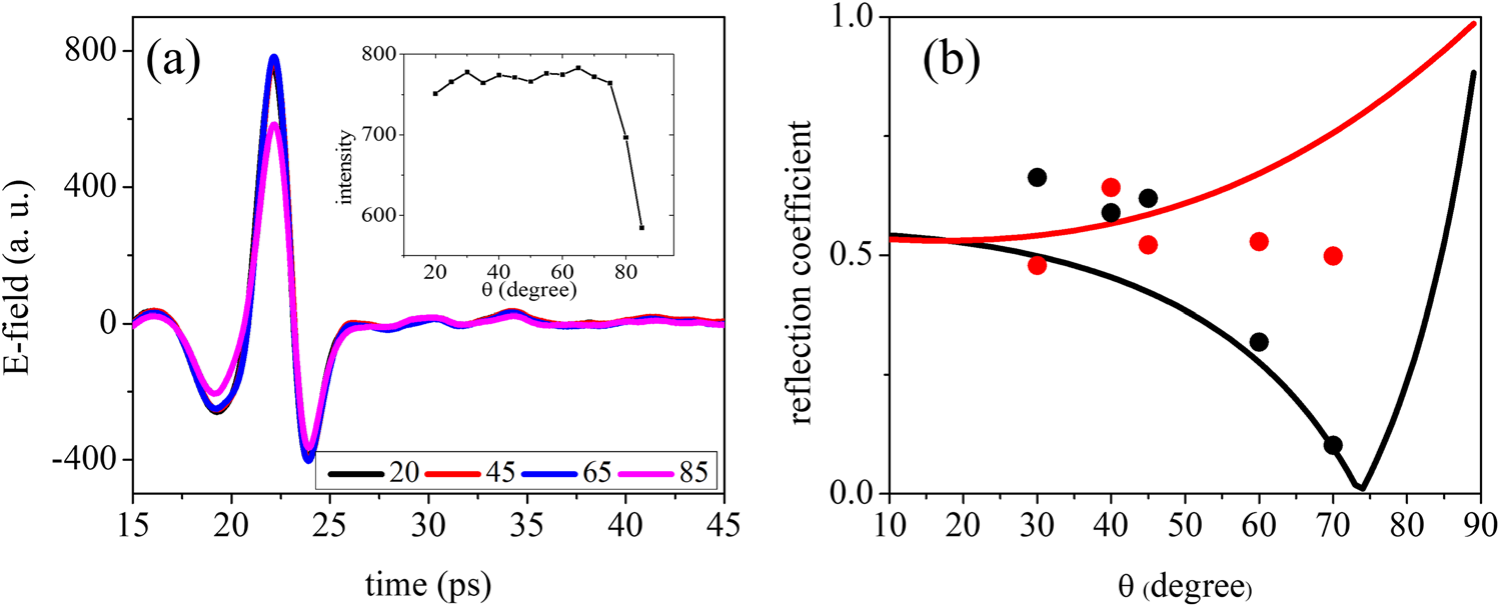
(**a**) Time profile of the electric field recorded by the THz-TDSE after the beam is reflected from the gold reflector at different angles. Signals all overlap but for $\theta = 85^{\circ }$. Inset shows the electric field peak intensity versus $\theta$. (**b**) Expected $r_{p}$ and $r_{s}$ behavior for high resistivity Silicon (black and red continuous curves respectively) in comparison with experimental values (black and red full circles) as a function of the incident angle for the uncalibrated setup at 0.4 THz.

(i) Although it is suggested to conduct ellipsometric measurements in the vicinity of the Brewster’s angle $\theta _{B}$ for maximum sensitivity^15^, it is wise to examine the detected THz signal at different incident angles upon reflection from a near perfect reflector (an un-coated gold reflector). Figure 2a displays the electric field time profile and the signal intensity recorded by our ellipsometer varying $\theta$, showing that the optimal range lies between $\theta =20^{\circ }$ (lower limit given by the geometric dimensions of the PCAs and the optical components) and $\theta =80^{\circ }$, above which the signal intensity suddenly drops. At large incident angles in fact the projection of the beam spot on the surface becomes too large and the polarization ellipse after the interaction with the sample starts exceeding the detector antenna aperture, with the result that not all the reflected beam is collected.
(ii) According to the definition of the Brewster’s angle of any material, near its value the amplitude of p polarization reaches its minimum, whereas s polarization monotonously increases^28^. For instance, since $\theta _{B}$ for Silicon in the THz range is $73.97^{\circ }$, we expect to observe the aforementioned behavior already approaching $70^{\circ }$. Figure 2b shows the quantities $r_{p}$ and $r_{s}$, before calibration, measured upon reflection from the Silicon sample surface at different incident angles, in comparison with the expected theoretical behavior. As it is evident from the graph, there is a substantial discrepancy that needs to be corrected using an appropriate calibration.
(iii) To this aim, we developed a method based on time domain analysis that can be used to set the best compensator azimuth angle ($\phi _{C}$) for a given incident angle in a very short period of time.

**Figure 3:**
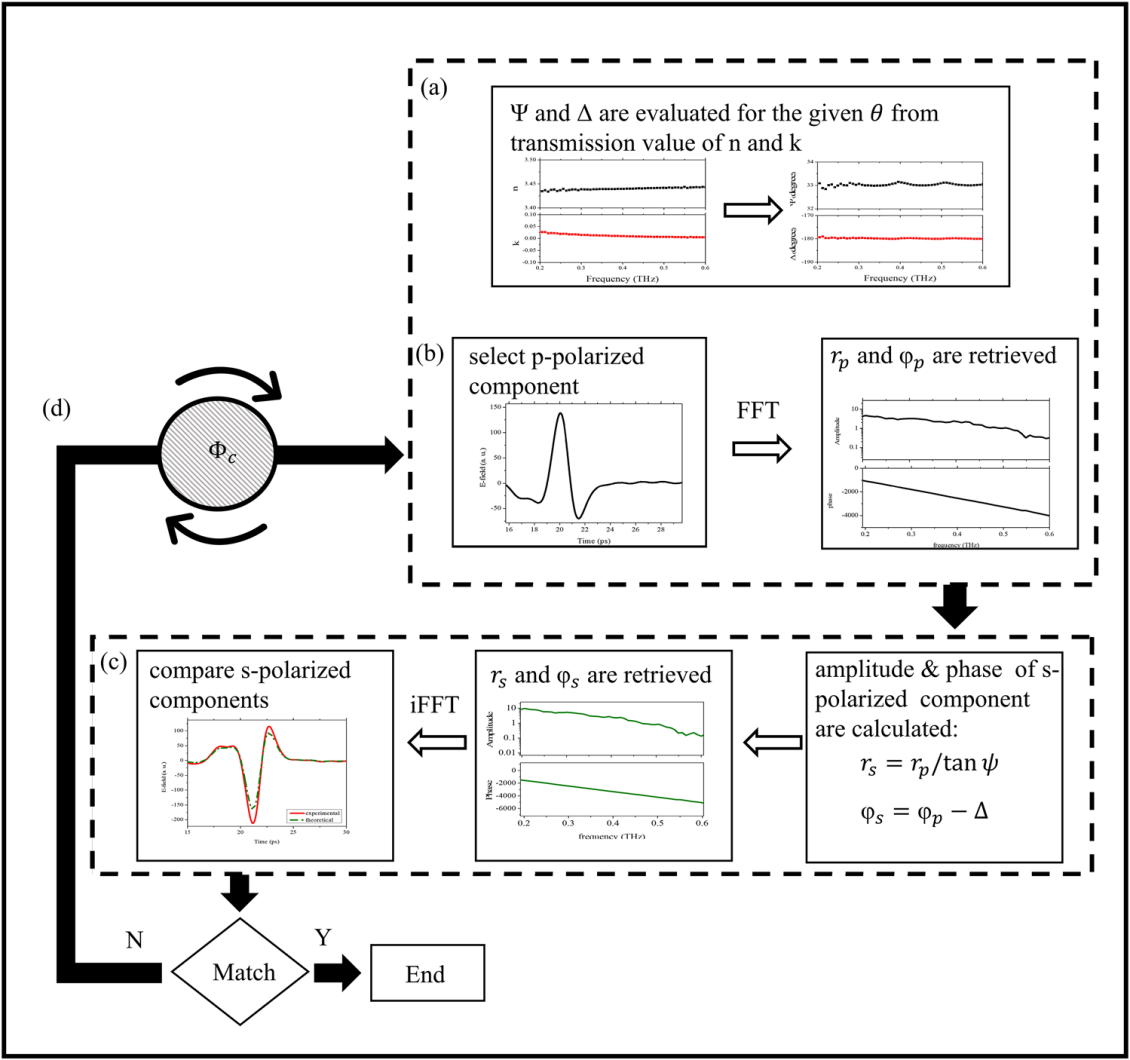
Flowchart of the ellipsometer calibration procedure.

As it appears in Eqs. (2) and (3), the ellipsometric angles $\Psi$ and $\Delta$ are easily calculated knowing the optical constants of a material. Subsequently, they can be used as input values for the calibration algorithm. For this reason the choice of an appropriate sample for the ellipsometer calibration is crucial. We chose a high resistivity single crystalline Silicon disk of radius 25 mm, since it is an homogeneous material with well-known properties in THz region. Moreover, its high surface quality (40-20 Scratch-Dig), parallelism ($< 2$ arcmin) and relatively large thickness (5 mm)) help in minimising or preventing surface scattering and backside reflections. The stepped logic sequence representing the calibration procedure is shown in the flowchart of Fig. 3.

First, the precise evaluation of the THz optical constants for the selected calibration sample is carried out using the same setup in transmission mode. Then, relying on Eqs. (2) and (3), from these measurements the related ellipsometric angles ($\Psi$ and $\Delta$) can be easily extracted (Fig. 3, step (a)). Selecting the p-polarised component of the electric field at a fixed incident angle and using FFT, its amplitude and phase as a function of frequency can be retrieved (Fig. 3, step (b)). From the previously determined $\Psi$ and $\Delta$ the expected amplitude and phase of the s-polarized component can be evaluated at any given $\theta$. The procedure is then completed by taking the inverse Fast Fourier Transform (iFFT) to determine the expected s-polarized signal in time domain and by comparing its temporal profile with the experimental one (Fig. 3, step (c)). At the end of the process, the azimuth angle $\phi _{C}$ of polariser C is changed clockwise or counter-clockwise till the best matching between experimental and theoretical s-polarized component of the electric field is achieved (Fig. 3, step (d)). The whole procedure outlined in the flowchart usually ends in less than 5 iterations per incident angle.

**Figure 4:**
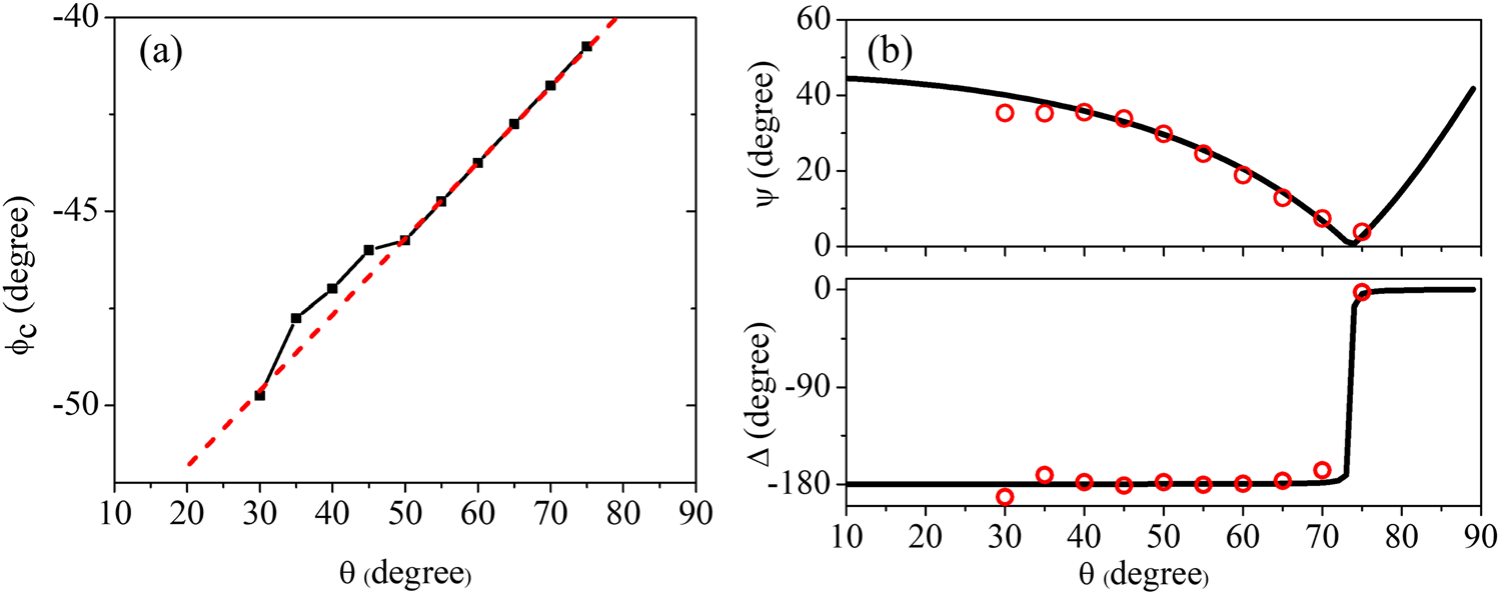
(**a**) Calibration curve for high resistivity monocrystalline Silicon. Values of $\phi _{c}$ are obtained for different $\theta$ using the algorithm-assisted method in time domain, (**b**) Comparison between experimental values (red full circles) and theoretical expectations (black continuous curves) of $\Psi$ and $\Delta$ vs $\theta$ in Silicon at 0.4 THz.

The final result of this time domain procedure is a calibration curve (Fig. 4a) that allows us to extract from the ellipsometric measurements, as shown in the following, the optical properties of a large number of materials at different incident angles with a great deal of confidence. It is worth to mention that the opposite procedure (selection of the s-polarised component and comparison between the experimental and theoretical p-polarised component) leads to exactly the same calibration curve.

From the graph in Fig. 4a, one can observe that the compensator azimuth angle doesn’t hold its “ideal” position $-45^{\circ }$ varying the incident angle. $\phi _{c}$ deviates more and more from this value as $\theta$ moves closer to $\theta _{B}$, since in that neighborhood the measurement accuracy enormously increases. The same happens—in the opposite direction—approaching grazing incidence, since the WGP has to compensate the distortion of the polarisation ellipse produced by non-ideal wavefronts. It is important also to highlight that the dependence $\phi _{c}(\theta )$ outlined in the plot is approximately linear, which allows to extrapolate with ease the values of the compensator azimuth angle for values of the incident angle outside the range of investigation.

In Fig. 4b we plot the experimental values of $\Psi$ and $\Delta$ obtained for Silicon at 0.4 THz for different incident angles and compare them with the theoretical expectations (continuous curves), verifying that they fully match the expected behavior^15^. While $\Psi$ monotonously decreases approaching the Brewster’s angle (since the p-polarised component goes to zero), $\Delta$ oscillates around -$\pi$ and suddenly changes only in close proximity of $\theta _{B}$ ($\approx 74^{\circ }$ for silicon).

This reveals a common disadvantage of the ellipsometry technique, that is a lower sensitivity than the one obtained from conventional transmission spectroscopy for the measurement of the extinction coefficient k. In general, ellipsometry is not a suitable characterization technique for the determination of k in materials having small absorption, preventing in such cases a reliable extraction of this parameter. Indeed, particularly in low loss samples it can be easily shown that variations in k (n) roughly affect $\Delta$ ($\Psi$) only^15^.

**Figure 5:**
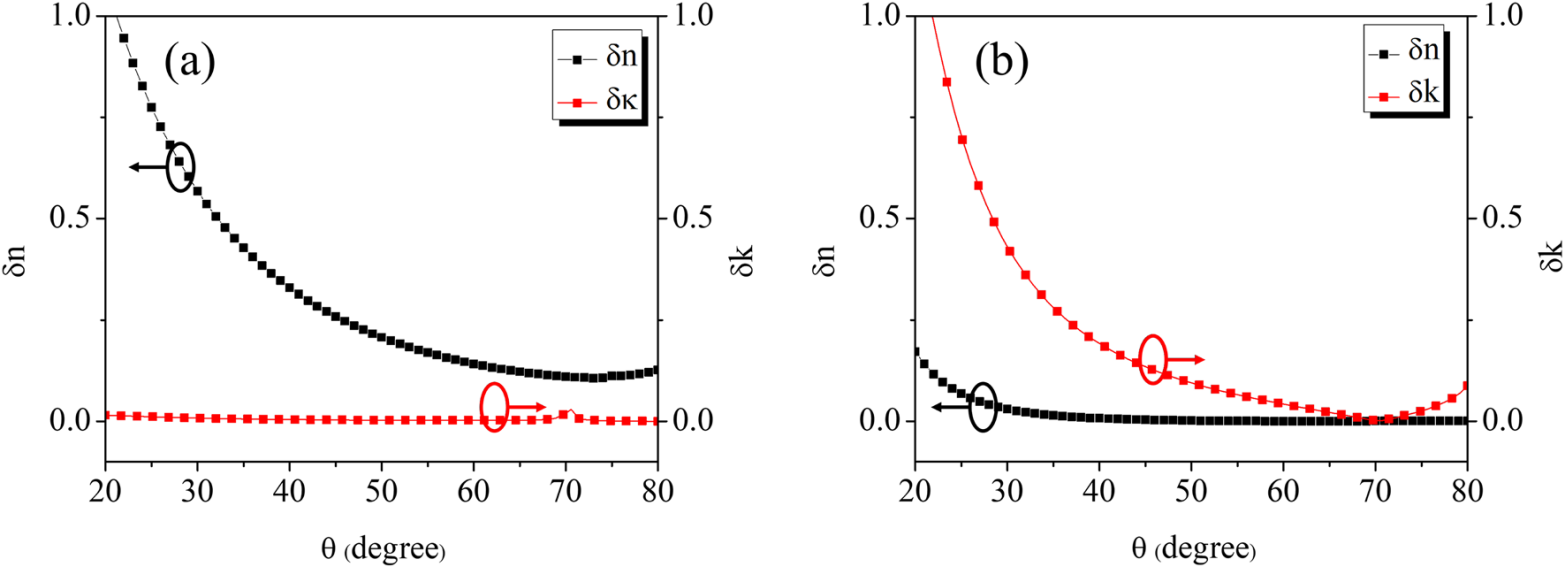
Deviations $\delta$n and $\delta$k of the Silicon complex refractive index estimated through ellipsometric angles measurements at 0.4 THz varying the signal incidence angle, with respect to reference values yielded using TDS. (**a**) $\Psi$ $\pm 1^{\circ }$, $\Delta$ constant. (**b**) $\Delta$ $\pm 1^{\circ }$, $\Psi$ constant.

Conversely, any deviation in the experimentally derived ellipsometric angles will produce a corresponding error in the extraction of the optical constants, that might be useful to examine better. Using Eqs. (2) and (3), we can empirically estimate what is the difference in the value of the optical constants retrieved for Si by the TDS and the TDSE techniques, with only a small error in the determination of $\Psi$ or $\Delta$. Figure 5a,b show how a $\pm 1^{\circ }$ deviation from the ideal value in $\Psi$ or $\Delta$ affects the accuracy of ellipsometric measurements. Results are obtained at 0.4 THz for different incident angles and plotted as $\delta$n=n$_{TDSE}$-n$_{TDS}$ and $\delta$k=k$_{TDSE}$-k$_{TDS}$. From the highly precise standard spectroscopy measurements, we use the value $n + ik = 3.430 + i0.009$. In the graph (a) on the left a $\pm 1^{\circ }$ variation is applied to $\Psi$, while $\Delta$ remains unchanged. To the contrary, graph (b) on the right shows results for $\Delta$ $\pm 1^{\circ }$ while keeping $\Psi$ constant. As expected, variations in $\Psi$ ($\Delta$) significantly affect only $\delta$n ($\delta$k). A common feature of the two graphs is that accuracy greatly increases near the Brewster’s angle of the sample, indicating the importance in ellipsometric measurements of a setup capable to operate in close proximity whatever is the material under test. For the evaluation of the extinction coefficient in particular, the precise measurement of $\Delta$ is mandatory, since ellipsometric data rapidly become unreliable as far as the incident angle deviates from $\theta _{B}$.

## Results and discussion

Once the calibration procedure is completed, we measure the dielectric response of a number of materials both in solid (quartz, sapphire, doped titanate) and liquid form (isopropyl and methyl alcohol, ultra-pure water). Solid samples are chosen to be homogeneous with an optically polished surface and thickness ranging approximately between 1 and 10 mm. Liquids are put in Petri dishes having 88 mm diameter and 14 mm depth to ensure a sufficiently large surface area and absence of back reflections. Each time-domain signal reflected from the sample is usually averaged over 1000 times, which takes less than a minute. Since we chose samples with quite different refractive index, the value of the respective Brewster’s angle varies between $57^{\circ }$ and $80^{\circ }$. This is a sufficiently broad range to check the robustness of the setup and the validity of the calibration procedure.

**Figure 6:**
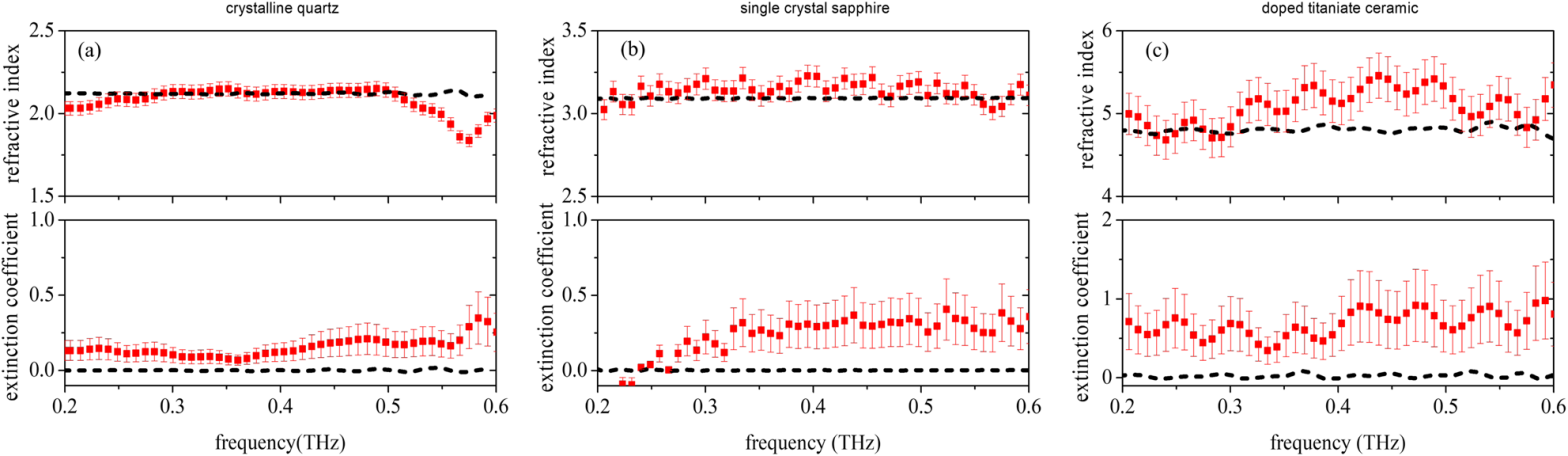
Complex refractive index of (**a**) crystalline z-cut quartz, (**b**) single crystal z-cut sapphire, and (**c**) doped titanate ceramic. Ellipsometric data are shown as red full circles, whereas black dashed lines represent the results of standard TDS measurements using the ellipsometer setup.

In Fig. 6 the results of ellipsometric measurements performed using the calibrated THz-TDSE setup on solid samples having thickness d are presented: (a) crystalline quartz, SiO$_{2}$ (d = 0.5 mm), (b) single crystal sapphire, Al$_{2}$O$_{3}$ (d = 8 mm), and (c) doped titanate ceramic, (Zr,Sn)TiO$_{4}$ (d = 1 mm). Both real and imaginary part of the complex refractive index as a function of frequency are shown as red full circles. Error bars are taken assuming a $\pm 1^{\circ }$ maximum deviation in the evaluation of the ellipsometric angles. Data are taken for an incident angle $\theta = 60^{\circ }$, in close proximity of the respective Brewster’s angle but for the ceramic titanate sample (c), where measurements due to the setup limits have been conducted at $\theta = 70^{\circ }$, quite far away from $\theta _{B}\approx 80^{\circ }$. Since both quartz and sapphire are birefringent materials, for (a) and (b) we chose z-cut crystals, that is samples oriented along the ordinary axis. Moreover, extra care is taken to ensure that the sample c-axis is perfectly orthogonal to the measurement plane. Under these conditions changes in polarisation produced by the difference between ordinary and extraordinary axis are minimized^29,30^ and can be neglected, since they are below the limit of detection of our setup.

In the case of the crystalline SiO$_{2}$, since it is a relatively thin sample, we observed the presence of backside reflections in the recorded s- and p-polarized beams. To reduce oscillations in the FFT spectra, we chose cutting and zero padding the time-domain signals. As for the single crystal sapphire, the significantly larger thickness obviates backside reflection effects, reducing on the opposite the signal-to-noise ratio in transmission spectroscopy. The pronounced Fabry-Perot oscillations observed in the ellipsometric data in the doped titanate are mostly due to the high refractive index combined with the limited thickness of the sample. The dashed-line curves represent the frequency dependence retrieved setting the THz ellipsometer in the transmission mode. We found that results obtained with TDSE and TDS experiments nicely match, with ellipsometric data only slightly overestimating the values of the extinction coefficient. They have also a good agreement with literature values^29,31,32^. For the doped titanate only, matching overall worsens because of the setup size constraints that prevent to perform measurements very close to $\theta _{B}$.

As already stated, the vertical design of our THz-TDSE shows a high potential for measuring liquids in a straightforward way, for the easiness in keeping a correct sample positioning with respect to the incident beam. As a matter of fact, studying liquids (especially polar ones) in the THz region is quite challenging since they are highly absorptive in this region of electromagnetic spectrum. In this respect, THz-TDSE ellipsometry promises to be a potentially disruptive technique for accurate measurements on liquids and more in general on biological (water-based) samples regardless of their thickness.

**Figure 7:**
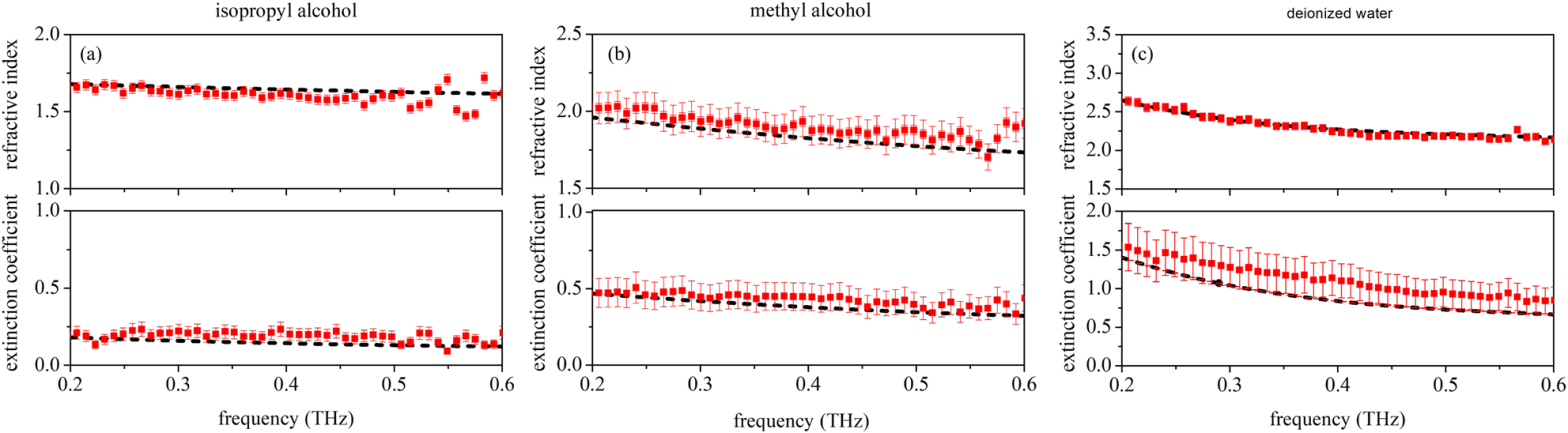
Complex refractive index of (**a**) isopropyl alcohol, (**b**) methyl alcohol, and (**c**) water. Ellipsometric data are shown as red full circles, whereas black dashed lines represent the results of standard TDS measurements using the ellipsometer setup.

Figure 7 reports the results of experiment performed on three different liquids: (a) isopropyl alcohol, 99.7 $\%$ purity, (b) methyl alcohol, 99.8 $\%$ purity, and (c) ultra-pure deionised (Milli-Q) water using THz-TDSE (red full circles) and standard spectroscopy (black dashed lines). All ellipsometric measurements have been carried out close to each sample Brewster’s angle ($\theta$ = $55^{\circ }$, $60^{\circ }$, and $65^{\circ }$ for isopropyl alcohol, methyl alcohol, and ultra-pure water, respectively). For transmission measurements on liquids, we use as sample holder a cuvette with $100~\mu m$ pool and quartz window plates (Hellma Analytics).

In liquids, the agreement between ellipsometric and standard spectroscopy measurements achieved in transmission mode is even better, and moreover data correspond with what reported in^33–35^. In particular, as far as the imaginary component of the complex refractive index is concerned, results confirm that THz-TDSE can be a useful tool and replace standard spectroscopy in all those situations where experiments in transmission mode are simply not possible.

In spite of the difficulties in the direct measurements of the ellipsometric angles with a high precision, we believe that results presented here show that THz-TDSE combined with a careful calibration procedure in time domain can give precious information with an unprecedented level of accuracy on the complex optical response of different classes of materials. For the extinction coefficient in particular, only a few experimental works to compare with are present in recent literature, reporting high mismatches^36^, or negative values^26^, or no values at all^24^.

## Conclusions

In this study, we have reported the design and fabrication of a customized fiber coupled THz ellipsometer with incident angle variable in a wide range, so that its configuration can be continuously changed from almost specular reflection to transmission mode. The calibration procedure, differently from other methods, operates in time domain and provides in a simple and fast way a significantly improved accuracy in the measurements of the complex refractive index.

To validate the system effectiveness, we have measured the optical constants of solid materials having from low (quartz) to high refractive index (doped titaniate) together with a number of polar fluids (water, isopropanol, and methanol) with relatively large absorption coefficients. Results on refractive index are in very good agreement with values obtained using the THz time domain setup in transmission mode. Moreover, using a time domain calibration approach, we have demonstrated that our setup is capable of measuring with a reasonable accuracy the extinction coefficient too, even if previous data have shown that ellipsometry is not a very suitable technique for extracting information on material with low absorption^24,26^. THz ellipsometry therefore represents a powerful, non destructive, contactless technique for the characterisation of lossy materials, having absorption coefficients above 100 cm$^{-1}$. Moreover, its self-reference feature makes THz-TDSE particularly versatile, robust and free from errors induced by imperfect evaluation or variations of the sample or substrate thickness. Therefore, it might lend itself well also to the characterisation of semiconductors with a large carrier concentration and conducting thin films and, in general, of other materials or systems where a high level of losses prevents using the transmission mode. In fact, standard spectroscopy measurements on highly absorptive samples are possible only for a limited thickness, usually much less than 1 mm.

Finally, since emitter and detector arms are designed to move in a plane perpendicular to the optical table, the setup is very suitable for measuring liquids and biological (water-based) samples for sensing applications. Because of its capability of extracting information on polarimetric properties, it might be also extremely useful in a near future as a novel characterisation tool for the development of THz devices^20^. Unfortunately, the present setup shows an inherent frequency band limitation caused by the required free space-to-fiber coupling to feed the PCAs, that unavoidably introduces extra losses and dispersion on the laser beam. Extending our approach to a full fiber coupled THz system will certainly lead to a much wider frequency range attainable using the time domain technique.

## References

[1] Zhang X-C, Xu J (2010). Introduction to THz Wave Photonics.

[2] Consolino L, Bartalini S, De Natale P (2017). Terahertz frequency metrology for spectroscopic applications: a review. J. Infrared Millimeter Terahertz Waves.

[3] Ho L, Pepper M, Taday P (2008). Signatures and fingerprints. Nat. Photonics.

[4] Zhong S (2019). Progress in terahertz nondestructive testing: a review. Front. Mech. Eng..

[5] Koral C (2020). Defects in the amorphous-crystalline evolution of gel-derived TiO_2_. J. Phys. Chem. C.

[6] Withayachumnankul W, Naftaly M (2014). Fundamentals of measurement in terahertz time-domain spectroscopy. J. Infrared Millimeter Terahertz Waves.

[7] Amoruso S (2020). All-carbon THz components based on laser-treated diamond. Carbon.

[8] Koral, C., Papari, G. & Andreone, A. THz spectroscopy of advanced materials. *Terahertz (THz), Mid Infrared (MIR) and Near Infrared (NIR) Technologies for Protection of Critical Infrastructures Against Explosives and CBRN*, 253–273 (2021).

[9] Naftaly M, Vieweg N, Deninger A (2019). Industrial applications of terahertz sensing: state of play. Sensors.

[10] Pavlou C (2021). Effective EMI shielding behaviour of thin graphene/PMMA nanolaminates in the THz range. Nat. Commun..

[11] Zhang L, Zhong H, Zhu D, Zuo J, Zhang C (2012). Feature extraction without phase error for THz reflective spectroscopy. Sci. China Inf. Sci..

[12] Tompkins H, Irene AE (2005). Handbook of Ellipsometry.

[13] Jellison G (1998). Spectroscopic ellipsometry data analysis: measured versus calculated quantities. Thin Solid Films.

[14] De Feijter J, Benjamins dJ, Veer F (1978). Ellipsometry as a tool to study the adsorption behavior of synthetic and biopolymers at the air–water interface. Biopolym. Orig. Res. Biomol..

[15] Fujiwara H (2007). Spectroscopic Ellipsometry: Principles and Applications.

[16] Baierl S (2016). Nonlinear spin control by terahertz-driven anisotropy fields. Nat. Photonics.

[17] Mukai Y, Hirori H, Yamamoto T, Kageyama H, Tanaka K (2014). Antiferromagnetic resonance excitation by terahertz magnetic field resonantly enhanced with split ring resonator. Appl. Phys. Lett..

[18] Sotome M, Kida N, Horiuchi S, Okamoto H (2014). Visualization of ferroelectric domains in a hydrogen-bonded molecular crystal using emission of terahertz radiation. Appl. Phys. Lett..

[19] Hofmann T, Herzinger C, Krahmer C, Streubel K, Schubert M (2008). The optical Hall effect. Physica Status Solidi (a).

[20] Karl N (2017). Characterization of an active metasurface using terahertz ellipsometry. Appl. Phys. Lett..

[21] Born M, Wolf E (2013). Principles of Optics: Electromagnetic Theory of Propagation, Interference and Diffraction of Light.

[22] Hofmann T (2010). Variable-wavelength frequency-domain terahertz ellipsometry. Rev. Sci. Instrum..

[23] Kühne P (2018). Advanced terahertz frequency-domain ellipsometry instrumentation for in situ and ex situ applications. IEEE Trans. Terahertz Sci. Technol..

[24] Neshat M, Armitage N (2013). Developments in THz range ellipsometry. J. Infrared Millimeter Terahertz Waves.

[25] Johs B (1993). Regression calibration method for rotating element ellipsometers. Thin Solid Films.

[26] Chen X, Parrott EP, Huang Z, Chan H-P, Pickwell-MacPherson E (2018). Robust and accurate terahertz time-domain spectroscopic ellipsometry. Photonics Res..

[27] Báez-Chorro MA, Vidal B (2019). Single trace terahertz spectroscopic ellipsometry. Opt. Express.

[28] Sassen K (1987). Polarization and Brewster angle properties of light pillars. J. Opt. Soc. Am. A.

[29] Grischkowsky D, Keiding S, Van Exter M, Fattinger C (1990). Far-infrared time-domain spectroscopy with terahertz beams of dielectrics and semiconductors. J. Opt. Soc. Am. B.

[30] Naftaly M, Gregory A (2021). Terahertz and microwave optical properties of single-crystal quartz and vitreous silica and the behavior of the boson peak. Appl. Sci..

[31] Davies CL, Patel JB, Xia CQ, Herz LM, Johnston MB (2018). Temperature-dependent refractive index of quartz at terahertz frequencies. J. Infrared Millimeter Terahertz Waves.

[32] Huang C-L, Weng M-H (2000). Liquid phase sintering of (Zr, Sn) TiO$$_4$$ microwave dielectric ceramics. Mater. Res. Bull..

[33] Balakrishnan J, Fischer BM, Abbott D (2009). Fixed dual-thickness terahertz liquid spectroscopy using a spinning sample technique. IEEE Photonics J..

[34] Huang S (2009). Improved sample characterization in terahertz reflection imaging and spectroscopy. Opt. Express.

[35] Zhou J (2019). Temperature dependent optical and dielectric properties of liquid water studied by terahertz time-domain spectroscopy. AIP Adv..

[36] Belyaeva A, Galuza A, Kolenov I, Mizrakhy S (2021). Developments in terahertz ellipsometry: Portable spectroscopic quasi-optical ellipsometer-reflectometer and its applications. J. Infrared Millimeter Terahertz Waves.

